# Patient-Reported Outcomes and Total Health Care Expenditure in Prediction of Patient Satisfaction: Results From a National Study

**DOI:** 10.2196/publichealth.4360

**Published:** 2015-09-23

**Authors:** Man Hung, Weiping Zhang, Wei Chen, Jerry Bounsanga, Christine Cheng, Jeremy D Franklin, Anthony B Crum, Maren W Voss, Shirley D Hon

**Affiliations:** ^1^ Department of Orthopaedics University of Utah Salt Lake City, UT United States; ^2^ Division of Public Health University of Utah Salt Lake City, UT United States; ^3^ School of Business University of Utah Salt Lake City, UT United States; ^4^ School of Medicine University of Utah Salt Lake City, UT United States; ^5^ Division of Epidemiology University of Utah Salt Lake City, UT United States; ^6^ College of Education University of Utah Salt Lake City, UT United States

**Keywords:** health care quality, value, expenditure, cost, medical outcomes, patient satisfaction, Medical Expenditure Panel Survey, patient-reported outcomes, Affordable Care Act, big data analytics

## Abstract

**Background:**

Health care quality is often linked to patient satisfaction. Yet, there is a lack of national studies examining the relationship between patient satisfaction, patient-reported outcomes, and medical expenditure.

**Objective:**

The aim of this study is to examine the contribution of physical health, mental health, general health, and total health care expenditures to patient satisfaction using a longitudinal, nationally representative sample.

**Methods:**

Using data from the 2010-2011 Medical Expenditure Panel Survey, analyses were conducted to predict patient satisfaction from patient-reported outcomes and total health care expenditures. The study sample consisted of adult participants (N=10,157), with sampling weights representative of 233.26 million people in the United States.

**Results:**

The results indicated that patient-reported outcomes and total health care expenditure were associated with patient satisfaction such that higher physical and mental function, higher general health status, and higher total health care expenditure were associated with higher patient satisfaction.

**Conclusions:**

We found that patient-reported outcomes and total health care expenditure had a significant relationship with patient satisfaction. As more emphasis is placed on health care value and quality, this area of research will become increasingly needed and critical questions should be asked about what we value in health care and whether we can find a balance between patient satisfaction, outcomes, and expenditures. Future research should apply big data analytics to investigate whether there is a differential effect of patient-reported outcomes and medical expenditures on patient satisfaction across different medical specialties.

## Introduction

Value-based health care has become a buzzword for health care reforms across the globe. In the United States, the Affordable Care Act has placed huge emphasis on health care value and quality [1]. Although it has yet to be clearly defined, health care quality is often linked to patient satisfaction [2-4]. Beginning on October 1, 2012, the Center for Medicare & Medicaid Services (CMS) has tied Medicare reimbursements with patient satisfaction, as measured by the Hospital Consumer Assessment of Healthcare Providers and Systems (CAHPS) survey. The CAHPS survey measures the following 5 aspects of patient satisfaction or perception of health care experiences: (1) access to care, (2) provider communication, (3) coordination of care, (4) shared decision-making, and (5) the office staff. Additionally, it contains a global rating item on patient satisfaction. The results of the patient satisfaction survey are estimated to put a hospital at risk of US $500,000 to US $850,000 on average for Medicare reimbursement [5]. The stakes are high with patient satisfaction, but how can we improve patient satisfaction and what are the predictors of patient satisfaction?

One question is "what constitutes value in health care?" Porter posited that health care value is linked to patient-reported outcomes (PROs) and expenditures [6]. PROs are patients’ self-reported health status based on their perceptions of their health conditions. PROs, in addition to information from clinical assessments, have become a critical component of medical assessment by directly receiving information from the patient’s perspective [7]. Some argue that understanding PROs is critical to understanding patient satisfaction [8]. PRO instruments, such as SF-12 v2, can measure various outcomes including physical health, mental health, and general health.

While PROs have become increasingly important in assessing health care value, the second part of the value equation is cost or expenditures in health care. Prior research has showed mixed findings between expenditures and patient satisfaction [9-12]. Fenton et al [11] found that higher inpatient utilization, lower emergency department utilization, higher total medical expenditures, higher prescription drug expenditures, and fewer emergency visits are associated with higher patient satisfaction. However, a review of the literature found no consistent relationship and the majority of studies only found a small association between expenditures and satisfaction [10].

Research has suggested that a number of sociodemographic characteristics correlate with patient satisfaction, but there is not a strong consensus [13]. Several studies demonstrated that older patients, patients with better functional status, and clearer communication from doctors are related to greater patient satisfaction [9,11,14-16]. Rahmqvist and Bara [17] also found that older patients were more satisfied than younger patients [17], yet critiques suggested that the magnitude of age as a predictor of satisfaction is small [18]. Additionally, greater patient satisfaction has been associated with lower levels of education and income [17-19]. However, Hall and Dornan [18] found no relationship among income, ethnicity, sex, or family size with patient satisfaction.

Although sociodemographics and other nonmodifiable characteristics may influence patient satisfaction, they are not very useful for implementing changes in health care. Modifiable characteristics such as patient outcomes and health care expenditures are more useful for attaining actionable changes and reform in health care, yet there is a shortage of research in this area. There is also a lack of longitudinal, national studies examining the relationship between PROs, medical expenditures, and patient satisfaction. This study aims to fill this gap by investigating this relationship using a longitudinal, nationally representative sample, adjusting for various sociodemographics and health-related characteristics.

## Methods

### Study Design

#### Overview

Data from the longitudinal study of adult respondents to the 2010-2011 longitudinal panel Medical Expenditures Panel Survey (MEPS) [20] served as a basis for the comprehensive assessment of health care value and health care quality in this study. The MEPS is a nationally representative survey, sponsored by the Agency for Healthcare Research and Quality, which measures access, use, and cost of health care services. The survey consists of 3 major components, namely, (1) the household, (2) the medical provider, and (3) insurance. The household component samples were drawn from the respondents to the National Health Interview Survey by the National Center for Health Statistics. This study utilized data from the household component and included respondents aged 18 years or older. A list of all the variables used in this study is shown in Multimedia Appendix 1, and Multimedia Appendix 2 contains all of the covariates included in the analyses. Capitalizing on MEPS’s longitudinal panel survey design, we sought to predict patient satisfaction from PROs and total health care expenditures, controlling for demographics, prior health status, and clinical characteristics.

#### Medical Expenditures

The MEPS collects data on various categories of medical expenditures such as prescription drugs, emergency patient visits, and inpatient hospital stays. The variable total expenditure is an aggregate of medical expenditures in various categories. In this study, we used total health care expenditures (EXP) from Year 1 (2010) as an independent variable.

#### Patient-Reported Outcomes

The MEPS also contains PROs data. Specifically, it contains data from the SF-12v2, which measures patients’ self-reported functional health status and well-being. The SF-12v2’s physical health component score (PCS), the mental health component score (MCS), and the general health perceptions (GH) score from Year 1 were also included as independent variables in this study. The PCS and MCS possible scores range from 0 (worst health) to 100 (best health). To put GH in the same direction as the PCS and MCS scoring, we reverse coded the original GH such that the possible scores range from 1 (worst health) to 5 (best health).

#### Patient Satisfaction

We used the CAHPS’s single-item global rating of satisfaction in Year 2 (2011) as a dependent variable. This global patient satisfaction item (SAT) reflects patients’ rating of their health care from all physicians and health care providers in the last 12 months when the patients were taking the survey. It is not a recall of a certain visit at a specific time (eg, at 3 month or 5 months ago), rather it represents a patient's average experience of all health care encounters within the last 12 months. The possible scores range from 0 (worst health care possible) to 10 (best health care possible). The survey was administered using computer-assisted personal interviewing technology.

#### Covariates

Based on a literature review, we identified a list of potential confounders from the MEPS data to be the covariates and adjusted for them when investigating the contribution of PROs and total health care expenditure to patient satisfaction. The list of potential confounders is included in Multimedia Appendix 2, which contains patients’ age, sex, education, race, ethnicity, income, insurance coverage, provider characteristics, prior health status, and different clinical conditions. The covariates selected for the regression analyses were confounders that showed significant associations with patient satisfaction.

### Data Analysis

The MEPS utilized a multistage, probability clustering sample design that enabled comprehensive examination of the US population. The sampling weight, stratification, clustering, multiple stages of selection, and disproportionate sampling from the MEPS were taken into account in the analyses so that the findings reported represented the entire US population. Sampling weight took into account the differential probability of sample selection and adjusted for nonresponses and missing data. Descriptive statistics were conducted to examine SAT, PCS, MCS, GH scores, EXP, and different service categories of medical expenditures across clinical conditions. We also performed descriptive statistics on all potential confounders and flagged those that had significant associations with patient satisfaction to be included as covariates in subsequent regression analyses.

To investigate the contribution of PCS, MCS, GH, and EXP on patient satisfaction, we conducted logistic regressions with adjustment of covariates and reported the odds ratio with associated 95% CI. We recoded the PROs, satisfaction, and expenditure variables into low/high SAT, PCS, MCS, GH, and EXP prior to running the logistic regressions. All statistical tests were two sided, were set at an alpha level of .05, and were conducted using SAS 9.3. Institutional review board (IRB) and/or ethics committee approval was not required as the MEPS data are freely available to the general public online.

## Results

### Demographics

The entire study sample consisted of adult participants (N=10,157), which represented 233.26 million adults (aged ≥ 18 years) in the United States. The mean age was 47 years (SE 0.28, range 18-85). Approximately 52% (121 million/233.26 million) were female and the majority were white (81%, 188 million/233.26 million) and black (12%, 27 million/233.26 million). About 15% (34 million/233.26 million) were Hispanic, approximately 34% (79 million/233.26 million) were unemployed, and nearly 12% (27 million/233.26 million) spoke non-English at home (Table 1).

**Table 1:** Demographics of the sample from MEPS (weighted N=233.26 million; unweighted N=10,157).

		Weighted		Unweighted	
Variable		n (millions)	%	n (millions)	%
**Sex**					
	Male	113	48.3	2385	72.3
	Female	121	51.7	916	27.7
**Race**					
	White	188	80.7	7060	69.5
	Black	27	11.7	1992	19.6
	American Indian/Alaskan native	2	0.7	84	0.8
	Asian	12	5.1	805	7.9
	Native Hawaiian/Pacific Islander	2	0.7	72	0.7
	Multiple races	3	1.1	144	1.4
**Ethnicity**					
	Hispanic	34	14.5	2475	24.4
	Black (not Hispanic or another race)	27	11.4	1950	19.2
	Asian (not Hispanic or another race)	12	5.0	794	7.8
	Other race (not Hispanic or another race)	161	69.1	4938	48.6
**Hispanic**					
	Hispanic	34	14.5	2475	24.4
	Not Hispanic	200	85.5	7682	75.6
**Employment status**					
	Employed	154	66.2	6465	63.9
	Not employed	79	33.8	3653	36.1
**Marital status**					
	Married	124	53.4	5172	50.9
	Widowed	15	6.3	612	6.0
	Divorced	26	11.3	1173	11.5
	Separated	5	2.3	312	3.1
	Never married	62	26.8	2888	28.4
**Currently smoke**					
	Yes	39	18.3	1732	18.5
	No	176	81.7	7606	81.5
**Language spoken at home**					
	English	205	88.3	8009	79.2
	Spanish	19	8.1	1555	15.4
	Another language	8	3.6	542	5.4
**Highest level of education**					
	High school or less with no degree	31	13.4	1986	20.0
	High-school graduate or GED	69	29.9	3239	32.5
	Associates degree, beyond college, but no degree	62	26.9	2359	23.7
	Bachelor's degree	45	19.4	1596	16.0
	Master's, PhD, or professional degree	24	10.4	777	7.8

This nationally representative adult population had a number of health conditions. About 32% (74.97 million/233.09 million) had high blood pressure and 5% (12.31 million/233.11 million) had coronary heart disease. Approximately 4% (8.52 million/233.20 million) had previously experienced a stroke. Almost 30% (70.59 million/233.07 million) had high cholesterol and 24% (56.34 million/233.18 million) had arthritis. There were 10% (23.48 million/233.20 million) diagnosed with cancer.

The sample population visited a variety of providers; however, most visited a general/family practice physician (71%, 60.69 million/86.00 million) and internal medicine physicians (20%, 17.10 million/86.00 million) within the last 12 months. About 68% (101.57 million/150.09 million) stated that their providers always showed respect for their treatment, whereas 3% (3.89 million/150.09 million) stated that their providers never showed respect. Almost 96% (161.35 million/168.25 million) indicated that their providers explained treatment options but 4% (6.90 million/168.25 million) did not. Approximately 67% (156.18 million/233.26 million) had private insurance, 18% (41.31 million/233.26 million) had public health insurance, whereas over 15% (35.77 million/233.26 million) were uninsured. A detailed breakdown of the number of responses for each variable is not provided here but can be obtained by contacting the authors.

### Medical Expenditures

The 2010 annual total health care expenditure in the US was over US $1.11 trillion and the average total health care expenditure per adult was US $4752.39 (Table 2). The total medical expenditures for emergency visits, hospital stay, dental care, and prescription drugs were US $7.17 billion, US $41.21 billion, US $62.87 billion, and US $252.45 billion, respectively. The US medical expenditures, by major service categories, by disease conditions are displayed in Table 2. Table 3 presents the descriptive statistics for medical expenditure, outcomes, and patient satisfaction.

**Table 2:** US medical expenditure by service area, by condition, per adult in 2010 (in US $).

		Median	Mean	SE	Minimum	Maximum
**Emergency visits**						
	High blood pressure	0	38.19	3.92	0	3406
	Coronary heart disease	0	60.95	11.80	0	1576
	Angina	0	77.89	16.52	0	900
	Stroke	0	56.73	11.30	0	1092
	Emphysema	0	60.23	14.10	0	701
	High cholesterol	0	36.29	4.89	0	4570
	Cancer	0	34.52	5.72	0	936
	Arthritis	0	42.72	5.41	0	4570
	Asthma	0	48.23	9.12	0	4570
	Hysterectomy	0	52.49	9.38	0	5424
	Heart attack	0	56.23	11.52	0	732
**Hospital stay**						
	High blood pressure	0	233.94	29.24	0	28,798
	Coronary heart disease	0	498.17	115.31	0	17,792
	Angina	0	674.49	190.66	0	17,792
	Stroke	0	480.89	123.35	0	17,792
	Emphysema	0	381.02	96.18	0	5155
	High cholesterol	0	228.66	27.37	0	17,792
	Cancer	0	229.43	41.66	0	8923
	Arthritis	0	227.38	27.28	0	17,792
	Asthma	0	183.19	33.72	0	9289
	Hysterectomy	0	214.13	25.81	0	7550
	Heart attack	0	572.91	178.98	0	17,792
**Dental care**						
	High blood pressure	0	235.39	20.11	0	15,692
	Coronary heart disease	0	252.75	68.19	0	10,289
	Angina	0	198.33	57.69	0	3514
	Stroke	0	180.72	38.08	0	3514
	Emphysema	0	250.03	75.47	0	5998
	High cholesterol	0	273.77	22.51	0	15,692
	Cancer	0	291.01	31.46	0	6192
	Arthritis	0	259.89	21.74	0	15,692
	Asthma	0	203.07	23.56	0	5998
	Hysterectomy	0	266.83	23.91	0	9674
	Heart attack	0	188.15	58.59	0	5998
**Prescription drugs**						
	High blood pressure	711.50	1934.43	82.76	0	50,667
	Coronary heart disease	2030.00	3209.79	246.04	0	23,355
	Angina	2080.50	3269.23	329.06	0	17,576
	Stroke	1459.00	2902.59	326.25	0	40,940
	Emphysema	2150.00	3292.43	334.82	0	15,071
	High cholesterol	779.00	1985.17	85.30	0	40,940
	Cancer	691.00	2153.63	180.58	0	37,509
	Arthritis	914.50	2178.59	100.20	0	50,667
	Asthma	571.50	1863.06	130.80	0	31,763
	Hysterectomy	826.50	1826.24	104.19	0	40,940
	Heart attack	1858.00	3141.72	326.99	0	23,355

**Table 3:** Descriptive statistics for medical expenditure, outcomes, and patient satisfaction.

Variables	Mean (SE)	95% CI
Physical health component score (PCS)	49.28 (0.16)	48.97-49.59
Mental health component score (MCS)	50.98 (0.14)	50.7-51.25
General health (GH)	3.55 (0.02)	3.52-3.58
Total health care expenditure (EXP), US $	4752.39 (145.43)	4465.61-5039.17
Patient satisfaction (SAT)	8.30 (0.03)	8.24-8.36

### Patient-Reported Outcomes

The mean score for PCS, MCS, and GH was 49.28 (SE 0.16), 50.98 (SE 0.14), and 3.55 (SE 0.02), respectively. These scores were significantly correlated with each other (PCS with MCS, ρ=.16, *P*<.001; PCS with GH, ρ=.96, *P*<.001; MCS with GH, ρ=.62, *P*<.001).

### Patient Satisfaction

As measured by CAHPS’s global rating, the mean patient satisfaction score across the national adult sample was 8.30 (SE 0.03). The following were some of the variables that were significantly associated with patient satisfaction: age (ρ=.25, *P*<.001), currently smoking (χ^2^_1_=29.7, *P*<.001), provider showed respect for treatment (χ^2^_3_=75.5, *P*<.001), and provider explained option to person (χ^2^_1_=21.0, *P*<.001). The complete list of all of the variables that were found to be associated with patient satisfaction is provided in Multimedia Appendix 2.

### Patient-Reported Outcomes, Medical Expenditures, and Patient Satisfaction

Total health care expenditure was negatively related to PROs; individuals who had lower physical function (ρ=−.43, *P*<.001), lower mental function (ρ=−.11, *P*<.001), and lower general health (ρ=−.51, *P*<.001) had higher total health care expenditures. After adjusting for covariates, we found that PROs and total health care expenditure were highly related to patient satisfaction (Table 4). Higher physical function, higher mental function, higher general health status, and higher total health care expenditure were associated with higher patient satisfaction. The odds of those who had high GH being satisfied were 6 times greater than those who had low GH (adjusted OR 5.98, 95% CI 2.95-12.12). High GH was defined as those who had excellent GH and low GH was defined as those who had poor or fair GH. There was more than a 2-fold difference in patient satisfaction between those who had high and low PCS (adjusted OR 2.54, 95% CI 1.36-4.72) and a 2-fold difference between those who had high and low MCS (adjusted OR 1.95, 95% CI 1.20-3.15). Those who had high EXP being satisfied were 3 times greater than those who had low EXP (adjusted OR 3.20, 95% CI 1.47-6.98). High PCS, MCS, and EXP were defined by those respondents who had ≥75 percentile of the scores, whereas low PCS, MCS, and EXP were defined by those with <25 percentile scores.

**Table 4:** Prediction of patient satisfaction.

Independent variables	n	Adjusted odds ratio^a^	95% CI
Physical health component score (PCS)	4782	2.54	1.36-4.72
Mental health component score (MCS)	4728	1.95	1.20-3.15
General health (GH)	3251	5.98	2.95-12.12
Total health care expenditure (EXP)	5082	3.20	1.47-6.98

^a^Covariates are listed in Multimedia Appendix 2.

## Discussion

### Principal Findings

The purpose of this study was to investigate the contribution of physical health, mental health, general health, and total health care expenditures to patient satisfaction. We found that higher scores of physical health, mental health, and general health were related to higher scores of patient satisfaction. Consistent with previous research, we found that some factors such as age and provider interactions could impact patient satisfaction. Additionally, total health care expenditure was associated with patient satisfaction. Taken together, findings from this study can help providers, payers, policy makers, and the general public better understand the relationship between PROs, health care expenditures, and satisfaction.

General health demonstrated the strongest relationship with patient satisfaction. After accounting for demographics and various factors, we see that greater patient satisfaction is directly related to greater physical and mental health, although the relationship is stronger with physical health. The argument that the relation between higher patient satisfaction and better health is an artifact of the tendency of healthy patients to be satisfied is not new [21]. This stresses the importance of controlling for prior health status as we did in this study and with the inclusion of prior clinical conditions into the model. Previous research found that a functional measure of health predicted greater satisfaction levels immediately following the medical treatment [9]. Differing research results regarding the impact of patient satisfaction on health have been partially attributed to differences in how risk or health have been assessed as well as the time frame as to when the satisfaction scores were gathered [22]. It may be that individuals who are in poor health have to visit a physician more often, increasing the likelihood of a bad or unsatisfactory visit. Prior experience with health care, along with age and mental status variables had been shown to impact patient expectations [23]. Our study emphasizes the importance of controlling for prior health status, prior health care experience, nonmodifiable characteristics, and patient expectations when assessing the effect of patient satisfaction.

Mental health often goes unnoticed or it is dismissed as someone having a bad day. Both identifying mental health issues and addressing problems require training that goes beyond that of a general physician. As a result, a patient or a provider often needs to raise a concern about mental health. A stigma still exists about discussing and treating mental health issues, which may lead patients to be less willing to discuss mental health conditions. Seeing a mental health specialist adds an additional cost for patients that they may want to avoid. Finally, patients might not even notice that they have mental health issues. These may be reasons why mental health is a weaker predictor of patient satisfaction than physical health.

A surprising finding was that total health care expenditures also had a significant relation to patient satisfaction. In a recent review of health care quality, only one third of the 61 studies reviewed found a positive association between higher spending and better health care quality [10], but did not address patient satisfaction directly as a measure of quality. While our findings add to the body of research that suggests an association between spending and patient satisfaction [11], it contradicts other research that states there is no association [9,24]. It is possible that initiatives that improve PROs and control cost at the same time contribute to patient satisfaction. Instead of raising health care expenditures, health policy makers and providers should attend to modifiable characteristics that are within their control such as providers showing respect and providers explaining treatment options to enhance PROs and thus, patient satisfaction.

### Limitations

We were limited by the measurement tools in the MEPS, thus limiting the data we could use to measure physical, mental, and general health. Newer instruments have been developed using advanced techniques and these instruments may have better psychometric properties than instruments included in MEPS. The MEPS was administered using computer-assisted personal interviewing technology and the results obtained from this mode of survey administration may be different from those obtained from other modes. Care needs to be taken when doing cross-comparisons of results from different modes in different studies. Furthermore, the definition of health care expenditures reflects the CMS’s definition but might not represent a complete assessment of medical expenditures. We were not able to look at diseases that were not included in MEPS. Even though we had adjusted for nonresponses and missing data in the sample, such adjustment could only be made to a certain degree. The adjustment might not be adequate in the event that nonrespondents differed from respondents. It is well-known that there could be a larger share of nonrespondents having severe illness, impairment, and dealing with poor social economic conditions on a daily basis. Additionally, the majority of the population is white, but within the past few years, the United States has seen a growth of racial and ethnic diversity. Therefore, our findings might not represent underrepresented minorities at this moment in time.

Finally, the concept of patient satisfaction lacks a clear connotation and definition in the literature. It has sometimes been considered as a component of consumer marketing [6] that measures important customer service qualities within the realm of health care. These qualities include effective provider communication, support from physicians, waiting time, fulfillment of patient requests, staff integrity, and shared decision making, and represent multiple dimensions of patient satisfaction. Together, these dimensions of patient satisfaction constitute a patient’s overall health care experience. It is this overall patient health care experience that this study used to define patient satisfaction. It is likely that we were not accounting for everything that comprises patient satisfaction. In the future it would be beneficial to collect different aspects of patient satisfaction data and examine them under a different lens.

### Future Research Directions

Future research should consider other modifiable characteristics that may influence patient satisfaction so that changes can be enacted. Often, underrepresented minorities have different experiences with health care than European Americans. Therefore, investigating their perspectives may illuminate group differences. Finally, future research should apply business intelligence or big data analytics to investigate whether there is a differential effect of PROs and medical expenditures on patient satisfaction across different medical areas.

### Conclusions

We found that PROs and total health care expenditure had a strong relationship with patient satisfaction. As more emphasis is placed on health care value and quality, this area of research will become increasingly needed. Critical questions should be asked about what we value in health care and whether we can find a balance between patient satisfaction, outcomes, and expenditures. These questions need to be asked to policy makers, physicians, patients, insurers, and the general public.

## Appendix

Multimedia Appendix 1 List of all MEPS variables examined in this study.

Multimedia Appendix 2 Association between patient satisfaction and potential confounders. Variables are rank listed with the strongest relations to patient satisfaction at the top and the lowest relations at the bottom.

## Abbreviations

CAHPS: Consumer Assessment of Healthcare Providers and Systems
CMS: Center for Medicare & Medicaid Services
EXP: total health care expenditure
GH: general health
MCS: mental health component score
MEPS: Medical Expenditures Panel Survey
PCS: physical health component score
PRO: patient-reported outcomes
SAT: patient satisfaction

## References

[1] Reineck LA, Kahn JM (2013). Quality measurement in the Affordable Care Act: a reaffirmed commitment to value in health care. Am J Respir Crit Care Med.

[2] Sequist TD, Schneider EC, Anastario M, Odigie EG, Marshall R, Rogers WH, Safran DG (2008). Quality monitoring of physicians: linking patients' experiences of care to clinical quality and outcomes. J Gen Intern Med.

[3] Kupfer JM, Bond EU (2012). Patient satisfaction and patient-centered care: necessary but not equal. JAMA.

[4] Glickman SW, Boulding W, Manary M, Staelin R, Roe MT, Wolosin RJ, Ohman EM, Peterson ED, Schulman KA (2010). Patient satisfaction and its relationship with clinical quality and inpatient mortality in acute myocardial infarction. Circ Cardiovasc Qual Outcomes.

[5] Buhlman N, Matthes N (2011). White Papers.

[6] Porter ME (2010). What is value in health care?. N Engl J Med.

[7] Deshpande PR, Rajan S, Sudeepthi BL, Abdul Nazir CP (2011). Patient-reported outcomes: a new era in clinical research. Perspect Clin Res.

[8] Manary MP, Boulding W, Staelin R, Glickman SW (2013). The patient experience and health outcomes. N Engl J Med.

[9] Jackson JL, Chamberlin J, Kroenke K (2001). Predictors of patient satisfaction. Soc Sci Med.

[10] Hussey PS, Wertheimer S, Mehrotra A (2013). The association between health care quality and cost: a systematic review. Ann Intern Med.

[11] Fenton JJ, Jerant AF, Bertakis KD, Franks P (2012). The cost of satisfaction: a national study of patient satisfaction, health care utilization, expenditures, and mortality. Arch Intern Med.

[12] Cohen JW, Monheit AC, Beauregard KM, Cohen SB, Lefkowitz DC, Potter DE, Sommers JP, Taylor AK, Arnett RH (1996). The Medical Expenditure Panel Survey: a national health information resource. Inquiry.

[13] Sitzia J, Wood N (1997). Patient satisfaction: a review of issues and concepts. Soc Sci Med.

[14] Hekkert KD, Cihangir S, Kleefstra SM, van den Berg B, Kool RB (2009). Patient satisfaction revisited: a multilevel approach. Soc Sci Med.

[15] Sitzia J, Wood N (1997). Patient satisfaction: a review of issues and concepts. Soc Sci Med.

[16] Stewart M, Brown JB, Donner A, McWhinney IR, Oates J, Weston WW, Jordan J (2000). The impact of patient-centered care on outcomes. J Fam Pract.

[17] Rahmqvist M, Bara A (2010). Patient characteristics and quality dimensions related to patient satisfaction. Int J Qual Health Care.

[18] Hall JA, Dornan MC (1990). Patient sociodemographic characteristics as predictors of satisfaction with medical care: a meta-analysis. Soc Sci Med.

[19] Young GJ, Meterko M, Desai KR (2000). Patient satisfaction with hospital care: effects of demographic and institutional characteristics. Med Care.

[20] (2011). Medical Expenditure Panel Survey.

[21] Covinsky KE, Rosenthal GE, Chren MM, Justice AC, Fortinsky RH, Palmer RM, Landefeld CS (1998). The relation between health status changes and patient satisfaction in older hospitalized medical patients. J Gen Intern Med.

[22] Manary MP, Boulding W, Staelin R, Glickman SW (2013). The patient experience and health outcomes. N Engl J Med.

[23] Bowling A, Rowe G, Lambert N, Waddington M, Mahtani K, Kenten C, Howe A, Francis SA (2012). The measurement of patients' expectations for health care: a review and psychometric testing of a measure of patients' expectations. Health Technol Assess.

[24] Fu AZ, Wang N (2008). Healthcare expenditures and patient satisfaction: cost and quality from the consumer's perspective in the US. Curr Med Res Opin.

