# Supplementary material for: Patient-Reported Outcomes and Total Health Care Expenditure in Prediction of Patient Satisfaction: Results From a National Study
Source: JMIR Public Health Surveill. 2015 Sep 23;1(2):e13. doi: 10.2196/publichealth.4360 (PMC4869209; doi:10.2196/publichealth.4360)
Supplement: Multimedia Appendix 1 [file publichealth_v1i2e13_app1.pdf]

Variable ID	Variable Name	% missing (based on 10,157 respondents)
1. AGE2X	AGE	0.0
2. SEX	GENDER	0.0
3. RACEX	RACE	0.0
4. HISPANX	HISPANIC ETHNICITY	0.0
5. MARRYY1X	MARITAL STATUS	0.0
6. HIBPDXY1	HIGH BLOOD PRESSURE DIAG	0.1
7. CHDDXY1	CORONARY HRT DISEASE DIAG	0.1
8. ANGIDXY1	ANGINA DIAGNOSIS	0.1
9. STRKDXY1	STROKE DIAGNOSIS	0.0
10. EMPHDXY1	EMPHYSEMA DIAGNOSIS	0.0
11. CHOLDXY1	HIGH CHOLESTEROL DIAGNOSIS	0.1
12. CANCERY1	CANCER DIAGNOSIS	0.0
13. ARTHDXY1	ARTHRITIS DIAGNOSIS	0.0
14. ASTHDXY1	ASTHMA DIAGNOSIS	0.0
15. HYSTER3	HAD A HYSTERECTOMY	47.4
16. BMINDX3	ADULT BODY MASS INDEX	3.8
17. ADSMOK2	CURRENTLY SMOKE	8.1
18. ADGENH2	HEALTH IN GENERAL	7.2
19. PCS2	PHYICAL COMPONT SUMMRY	7.0
20. MCS2	MENTAL COMPONT SUMMRY	7.0
21. ADINSA2	DO NOT NEED HEALTH INSURANCE	8.5
22. ADINSB2	HEALTH INSURANCE NOT WORTH COST	9.0

23. ADOVER2	CAN OVERCOM ILLS WITHOUT MED HELP	8.7
24. LANGHM2	LANGUAGE SPOKEN MOST IN HOME	0.5
25. PROVTY2	PROVIDER TYPE	29.9
26. TYPEPE2	TYPE OF PROVIDER	67.4
27. LOCATN2	LOCATION	30.0
28. HSPLAP2	IS PROVIDER HISPANIC OR LATINO	68.0
29. WHITPR2	IS PROVIDER WHITE	68.2
30. BLCKPR2	IS PROVIDER BLACK/AFRICAN AMER	68.2
31. ASIANP2	IS PROVIDER ASIAN	68.2
32. NATAMP2	IS PROVIDER NATIVE AMERICAN	68.2
33. PACISP2	IS PROVIDER OTH PACIFIC ISLNDR	68.2
34. OTHRCP2	IS PROVIDER SOME OTHER RACE	68.5
35. GENDRP2	IS PROVIDER MALE OR FEMALE	67.5
36. RESPCT2	PROVIDER SHOWS RESPECT FOR TREATMENTS	39.2
37. EXPLOP2	PROVIDER EXPLAINS OPTIONS TO PERSON	32.3
38. LANGPR2	PROVIDER SPEAKS PERSON'S LANGUAGE	95.5
39. INDCAT2	INDUSTRY GROUP	92.2
40. OCCCAT2	OCCUPATION GROUP	92.2
41. TTLPY1X	PERSON'S TOTAL INCOME	0.0
42. INSCOVY1	HEALTH INSURANCE COVERAGE INDICATOR	0.0
43. TOTEXPY1	TOTAL HEALTH CARE EXP	0.0

44. OPTOTVY1	# OUTPATIENT DEPT PROVIDER VISITS	0.0
45. OPDRVY1	# OUTPATIENT DEPT PHYSICIAN VISITS	0.0
46. ERDEXPY1	TOTAL EMERGENCY ROOM DR EXP	0.0
47. IPDEXPY1	TOTL HOSP STAZ DR EXP	0.0
48. DVTEXPY1	TOTAL DENTAL CARE EXP	0.0
49. RXEXPY1	TOTAL RX EXP	0.0
50. ADHECR4	RATING OF HEALTH CARE	41.4
51. EMPST2	EMPLOYMENT STATUS	0.4
52. EDRECODE	EDUCATION	2.0
53. MIDXY1	HEART ATTACK (MI) DIAG (>17)	0.0
