# Supplementary material for: Patient-Reported Outcomes and Total Health Care Expenditure in Prediction of Patient Satisfaction: Results From a National Study
Source: JMIR Public Health Surveill. 2015 Sep 23;1(2):e13. doi: 10.2196/publichealth.4360 (PMC4869209; doi:10.2196/publichealth.4360)
Supplement: Multimedia Appendix 2 [file publichealth_v1i2e13_app2.pdf]

Variable ID	Potential Confounder Name	Spearman Correlation (*P* value)	Chi-square (*P* value)
1. AGE2X	Age*	.25 (<.001)	
2. OPTOTVY1	# Outpatient Dept Provider Visits*	.14 (<.001)	
3. OPDRVY1	# Outpatient Dept Physician Visits*	.09 (<.001)	
4. BMINDX3	Adult Body Mass Index*	.07 (<.001)	
5. OPTOTVY1	# Outpatient Dept Provider Visits*	.14 (<.001)	
6. OPDRVY1	# Outpatient Dept Physician Visits*	.09 (<.001)	
7. TTLPY1X	Person's Total Income*	.08 (<.001)	
8. INSCOVY1	Health Insurance Coverage*		278.78 (<.001)
9. CHOLDXY1	High Cholesterol*		273.10 (<.001)
10. HIBPDXY1	High Blood Pressure*		272.52 (<.001)
11. ARTHDXY1	Arthritis*		211.76 (<.001)
12. ADINSA2	Do Not Need Health Insurance*		206.82 (<.001)
13. ADINSB2	Health Insurance Not Worth Cost*		204.11 (<.001)
14. ADOVER2	Can Overcome Ills Without Med Help*		179.25 (<.001)
15. MARRYY1X	Marital Status*		162.27 (<.001)
16. LANGHM2	Language Spoken Most in home*		120.96 (<.001)
17. CANCERY1	Cancer *		119.73

			(<.001)
18. SEX	Gender*		105.06 (<.001)
19. HYSTER3	Hysterectomy*		98.89 (<.001)
20. CHDDXY1	Coronary Heart Disease*		92.56 (<.001)
21. EMPST2	Employment Status*		86.15 (<.001)
22. RESPCT2	Provider Shows Respect*		75.58 (<.001)
23. RACETHNX	Ethnicity*		68.69 (<.001)
24. PROVTY2	Provider type*		55.39 (<.001)
25. MIDXY1	Heart Attack*		41.10 (<.001)
26. ANGIDXY1	Angina*		35.92 (<.001)
27. RACEX	Race*		32.90 (<.001)
28. EDRECODE	Education*		34.27 (0.005)
29. ADSMOK2	Currently Smoke*		29.73 (<.001)
30. TYPEPE2	Type of Provider		27.87 (.180)
31. EMPHDXY1	Emphysema*		23.25 (<.001)
32. OCCCAT2	Occupation Group*		22.07 (.009)

33. EXPLOP2	Provider Explains Options*		21.08 (<.001)
34. INDCAT2	Industry Group		20.20 (.124)
35. LOCATN2	Location of Provider*		18.88 (<.001)
36. STRKDXY1	Stroke*		15.31 (<.001)
37. ASTHDXY1	Asthma*		9.39 (.002)
38. HSPLAP2	Hispanic or Latino Provider*		8.36 (.004)
39. WHITPR2	White Provider*		7.77 (.005)
40. ASIANP2	Asian Provider*		4.76 (.029)
41. BLCKPR2	Black Provider		2.05 (.152)
42. GENDRP2	Provider Gender		1.33 (.248)
43. OTHRCP2	Provider is Some Other Race		1.08 (.298)
44. LANGPR2	Provider Speaks Person's Language		0.18 (.673)
45. PACISP2	Pacific Islander Provider		0.17 (.685)
46. NATAMP2	Native American Provider		0.09 (.926)

*Significant predictors of patient satisfaction and were the covariates used for prediction of patient satisfaction.
